# Experimental investigation on the geometry of GHZ states

**DOI:** 10.1038/s41598-017-13124-6

**Published:** 2017-10-16

**Authors:** Gonzalo Carvacho, Francesco Graffitti, Vincenzo D’Ambrosio, Beatrix C. Hiesmayr, Fabio Sciarrino

**Affiliations:** 1 Dipartimento di Fisica, Sapienza Università di Roma, I-00185 Roma, Italy; 2 University of Vienna, Faculty of Physics, Boltzmanngasse 5, 1090 Vienna, Austria

## Abstract

Greenberger-Horne-Zeilinger (GHZ) states and their mixtures exhibit fascinating properties. A complete basis of GHZ states can be constructed by properly choosing local basis rotations. We demonstrate this experimentally for the Hilbert space ${{\mathbb{C}}}_{2}^{\otimes 4}$ by entangling two photons in polarization and orbital angular momentum. Mixing GHZ states unmasks different entanglement features based on their particular local geometrical connectedness. In particular, a specific GHZ state in a complete orthonormal basis has a “*twin*” GHZ state for which equally mixing leads to full separability in opposition to any other basis-state. Exploiting these local geometrical relations provides a toolbox for generating specific types of multipartite entanglement, each providing different benefits in outperforming classical devices. Our experiment investigates these GHZ’s properties exploiting the HMGH framework which allows us to study the geometry for the different depths of entanglement in our system and showing a good stability and fidelity thus admitting a scaling in degrees of freedom and advanced operational manipulations.

## Introduction

Entanglement is a fundamental concept of quantum theory and lies at the heart of many key discoveries, ranging from quantum teleportation^1^ to quantum dense coding^2^, quantum computation^3–5^, and quantum cryptography^6,7^. Entanglement is not limited to distinguishable physical particles but exhibits itself also between different degrees of freedom^8–11^. Mathematically speaking, a physical system can be separable or entangled with respect to a chosen factorization of the tensor algebra which describes the quantum state. Usually, the experimental setup fixes the factorization and applying local unitaries does not change the entanglement properties. While for bipartite systems the query of separability is straightforward, namely the state is separable with respect to a particular bipartition or not, this concept is more complicated for multipartite systems.

States that are not even bi-separable with respect to all possible bipartitions are called genuine multipartite entangled states. These entangled states are of special interest since they are the extreme version of entanglement, that is all subsystems contribute to the shared entanglement feature^12–14^. Among this class there are further refinements due to very distinct physical properties, useful examples are the Greenberger-Horne-Zeilinger (GHZ) states^15^, the graph states, the *W*-states or Dicke-states.

Here we focus on GHZ states by employing two physical photons for which we consider the polarization degree of freedom and a two-dimensional subspace of the orbital angular momentum (OAM) degree of freedom for each photon^16^. Thus we explore a 16 dimensional Hilbert space with the structure ${{\mathbb{C}}}_{2}^{\otimes 4}$. In this case, one has three different “depth” of entanglement: the state can be tri-separable, bi-separable or genuine multipartite entangled between the different subsystems. We show how these different types of entanglement can be detected via specific non-linear entanglement witnesses. Moreover, we show how mixing states with different local information can be utilized to design states with specific entanglement properties. In detail, mixing two GHZ states does not result always in states with same properties concerning entanglement. Controlling the different types of entanglement properties of the finally generated state will be the key for interesting applications. Alternatively, from the theoretical perspective it is also interesting to ask what is the minimum number of pure states in a convex combination needed for a state to have specific properties concerning entanglement^17,18^.

### Multipartite Entanglement

In this work we focus on four-qubit GHZ states (which is identical in this case with a graph state)^19^, having e.g. the form

1 $$|GH{Z}_{0000}\rangle =\frac{1}{\sqrt{2}}\{\mathrm{|0000}\rangle +\mathrm{|1111}\rangle \}\mathrm{.}$$

**Figure 1:**
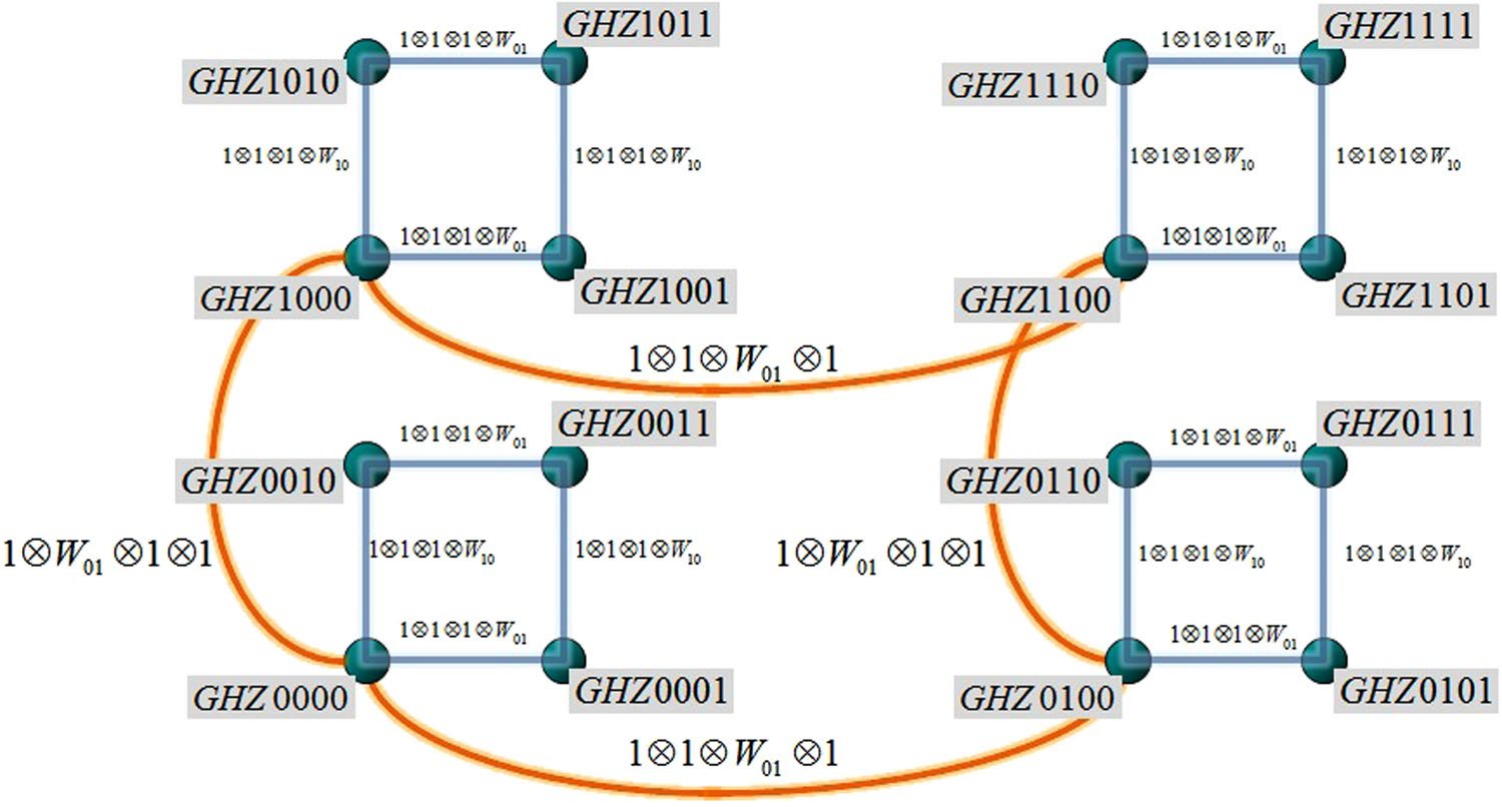
GHZ basis geometry. By applying the Weyl operators ${W}_{k,l}$ (Pauli’s operators) to the fourth subsystem it is possible to reach each quadrant’s vertex. In order to move horizontally/vertically from one quadrant to another one, it is necessary to apply a specific Weyl operator to the second, third subsystem or both subsystems^20^. Each GHZ state representation has a *twin* state, such that their equal mixtures looses all entanglement properties, in strong contrast to an equal mixing to any other GHZ state in the set.

By using a construction based on a minimal specific set of local basis rotations^20^ we can obtain the remaining 15 orthogonal basis states, e.g. $|GH{Z}_{i,j,k,l}\rangle ={\mathbb{1}}\otimes {W}_{\mathrm{0,}i}\otimes {W}_{\mathrm{0,}j}\otimes {W}_{k,l}|GH{Z}_{0000}\rangle$, where the Weyl operators *W* correspond in our case to the unity operator and the Pauli matrices (*W*_0,0_ = ${\mathbb{1}}$, ${W}_{\mathrm{0,1}}=X$, ${W}_{\mathrm{1,0}}=Z$, ${W}_{\mathrm{1,1}}=iY$). The construction procedure reveals how the states within a complete basis set relate by local unitary transformations. Starting with a seed state, e.g. $|GH{Z}_{0000}\rangle$ and by applying one of the three Pauli matrices to the fourth subsystem, we can obtain the other three mutually orthogonal GHZ states. How can we obtain the remaining 16 − 4 basis states? We have exploited all possible rotation with respect to the fourth subsystem, therefore we have to exploit another subsystem, e.g. the third one. However, certainly not all Weyl operators will lead to GHZ states that are orthogonal to the first four ones, indeed there is only one solution. Applying this specific Weyl operator, in our case *W*_0,1_, to the third subsystem we obtain a GHZ state mutually orthogonal to the four other ones. Now we can rotate again locally in the forth system, all these GHZ state are mutually orthogonal. Proceeding by applying in the second subsystem a specific Weyl operator (in our case *W*_0,1_) and again locally rotating in the fourth system we obtain a third set of four GHZ states that are mutually orthogonal to all other GHZ states. How can we obtain the remaining four GHZ states? It can be done by a combination of rotating in the second and third system via *W*_0,1_, respectively. Thus, our 16 GHZ basis states can be grouped into four with respect to a partition into the four subsystems. An experimenter having access to only one subsystem can obtain only a set of four GHZ basis states. To obtain a further set of four GHZ basis states one needs to have access to another subsystem. This is also visualized in Fig. 1.

Of course there is a democracy between any representation of a GHZ state (physics does not depend on the basis choice), however, and this is what we want to demonstrate, in the case we are superposing or mixing these different mutually orthogonal GHZ states, the properties concerning entanglement *do depend* on the specific local connection. Differently stated, we can exploit this local information to generate a state with specific properties with respect to entanglement. An experimenter has to take notice of the local information specific to his/her setup, this is what we will demonstrate experimentally by exploiting the quantum entanglement between two photons that are both in an internal entanglement between polarization and OAM degree of freedom a form of entanglement between two complex vectorial fields^11^.

The first thing to note is that when we mix two GHZ states of a complete basis set, the resulting state is still genuine multipartite entangled except when these states are equally mixed. In equal mixtures we still have two distinct categories:

*Type I (“twin” GHZ states)*: The resulting mixed state is fully separable.

*Type II (“un-twin” GHZ states)*: The resulting mixed state is entangled, though no longer genuine multipartite entangled, but still tripartite entangled.

Indeed *type I* states occur only for a single mixture, namely if one has chosen one GHZ state in the set there exists exactly one which erases the entanglement property, a “*twin*” GHZ state. This is immediately clear when considering the state defined in Eq. ((1)) and the one with a relative minus sign in the superposition. An equal mixture leads to zero off diagonal elements of the corresponding density matrix and, consequently, to a product state. Obviously, in all other cases we have four non-zero off-diagonal elements for which it is not straightforward to detect their separability properties. For that we exploit the HMGH framework^21^ providing a set of nonlinear witnesses for detecting *k*-separability. For a given matrix *ρ* to be *k*-separable the functions ${I}_{k}(\rho )$ (See appendix) have to be lower or equal zero, consequently a positive value detects *k*-inseparability.

For GHZ states the criterion *I*_2_ turns out to be optimal, namely the maximal value can be reached ${I}_{2}(|GHZ\rangle )=1$, whereas it is zero for any four-qubit Dicke-state with one excitation and $\frac{1}{2}$ for any four-qubit Dicke-state with two excitations (both states are known to be genuine multipartite entangled). Differently stated, *I*_2_ can be turned into an optimal witness for detecting the GHZ-type entanglement of a genuinely multipartite entangled state. For our purpose, the linearized version of this witness *I*_2_ denoted as ${\tilde{I}}_{2}$ is sufficient due to the high symmetry of the considered states and allows us to significantly reduced the amount of measurements to perform. However, note that for the other witnesses *I*_3,4_ we apply the non-linearized versions. Written in Pauli’s operators the linear witness detecting genuine multipartite entanglement becomes

2 $$\begin{array}{c}{\tilde{I}}_{2}(\rho )=\frac{1}{8}\langle XXXX-YYXX-YXYX-XYYX\\ \quad \quad \quad \,{-XXYY-XYXY-YXXY+YYYY\rangle }_{\rho }\\ \quad \quad \quad \,-\frac{1}{8}\langle 7{\mathbb{1111}}-ZZ{\mathbb{11}}-Z{\mathbb{11}}Z-Z{\mathbb{1}}Z{\mathbb{1}}\\ \quad \quad \quad \,{-{\mathbb{11}}ZZ-{\mathbb{1}}Z{\mathbb{1}}Z-{\mathbb{1}}Z{\mathbb{1}}Z-ZZZZ\rangle }_{\rho }\end{array}$$

where we used the abbreviation *XXXX* for *X* ⊗ *X* ⊗ *X* ⊗ *X* and so on. ${\tilde{I}}_{2}(\rho )$ detects genuine multipartite entanglement if it is greater than zero and gives the maximal value (equal to one) only for the GHZ state in the representation of Eq.((1)) (by exploiting local unitary operations the criterion can be made optimal for any basis representation of the GHZ state).

In the following we describe the production of all orthogonal basis states and prove the genuine multipartite entanglement property by the above introduced criteria via different methods. Finally we discuss how the entanglement properties change in the case of mixed GHZ states.

### Experimental generation of GHZ states

**Figure 2:**
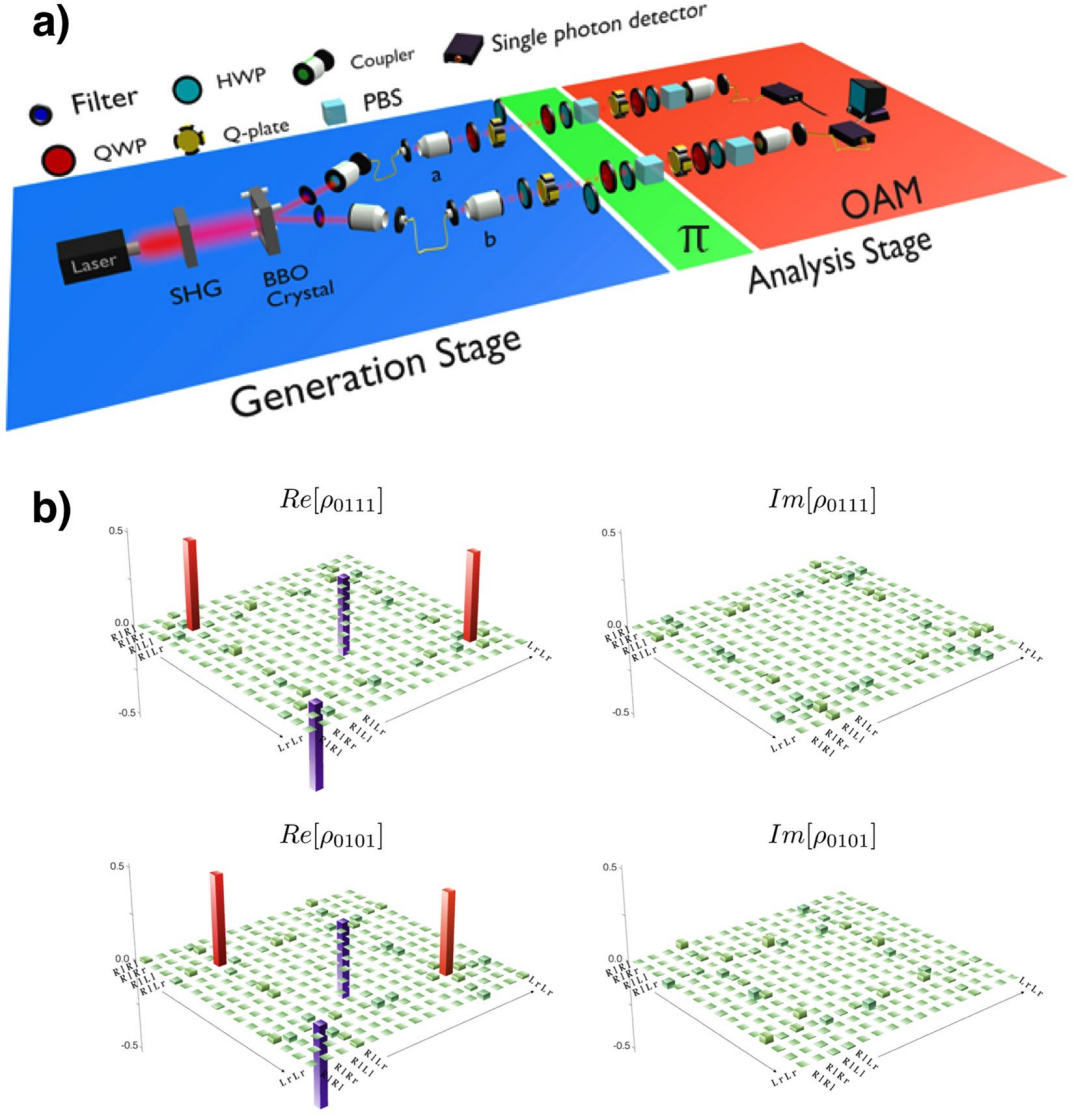
Experimental setup and generated states. (**a**) Experimental setup for generation and analysis of GHZ-states. In the generation stage the state of each of two entangled photons (a and b) is locally manipulated via QWP, HWP and q-plates with settings according to the particular GHZ state to be prepared. The analysis stage is divided in two sections one for the polarization analysis *π* and the other for OAM analysis. The polarization analysis is performed by using a stage composed of QWP, HWP and PBS. The OAM analysis requires a q-plate to transfer the information encoded in the OAM space to the polarization degree of freedom which can be then analyzed by means of the same kit used in the *π*-section. After the analysis both photons are sent to single mode fibers connected to single photon detectors. (**b**) Experimental density matrices of two of the generated states (*ρ*_0111_ and *ρ*_0101_). Real and imaginary parts of the experimental density matrices are reconstructed via full quantum state tomography.

GHZ states can be generated with different physical systems^22–26^. Here we generate photonic four-qubit GHZ states by entangling polarization and OAM within each photon of an entangled photon pair. To this end we exploit the q-plate^27,28^, a birefringent slab with a suitably patterned transverse optical axis and a topological singularity at its center. Such device entangles or disentangles the OAM with the polarization for each photon. The experimental setup is shown in Fig. 2a.

The pump laser (wavelength *λ* = 397.5 nm) is produced by a second harmonic generation (SHG) process from a Ti:sapphire mode-locked laser with a repetition rate of 76-MHz. Type II spontaneous parametric down conversion (SPDC) in a *β*-barium borate (BBO) crystal is exploited to generate photon pairs entangled in polarization. These photons (*λ* = 795 nm) are filtered in the wavelength and spatial modes by using filters with ${\rm{\Delta }}\lambda =3$ nm and single-mode fibers, respectively. The resulting state can then be written in the polarization and OAM basis by

3 $$|{\psi }^{-}\rangle =\frac{1}{\sqrt{2}}\{{|R,0\rangle }_{a}{|L,0\rangle }_{b}-{|L,0\rangle }_{a}{|R,0\rangle }_{b}\},$$

where $|R,\ell \rangle$ $(|L,\ell \rangle )$ denotes a photon with circular right (left) polarization and carrying $\ell \hslash$ of OAM and the subscripts *a*, *b* refers to the two different photons. Each photon is sent to a q-plate whose action is given by

4 $$\begin{array}{l}|R,\,0\rangle \to |L,r\rangle \\ |L,\,0\rangle \to |R,l\rangle \end{array}$$

where, for uniformity of notation, we wrote *r* (*l*) to indicate OAM eigenstates with $\ell =-1\,(+\mathrm{1)}$. More details on the general transformation that can be operated by a q-plate can be found in^29^. As a consequence the state ((3)) is transformed into a GHZ state, $|GH{Z}_{0101}\rangle =1/\sqrt{2}(|RlLr\rangle -|LrRl\rangle )$ (omitting the photon label subscripts). The two first qubits represent the polarization and OAM degrees of freedom for one photon, whereas the third and fourth qubits represent the polarization and OAM degrees of freedom for the second photon. By applying specific local transformations to *GHZ*_0101_ using half wave plates (HWP) and quarter wave plates (QWP) we obtain any other GHZ state of a complete set of four-qubits GHZ states. After this stage, each photon is analyzed in the polarization and OAM degrees of freedom. The polarization-analysis stage is composed of QWP, HWP and polarizing beam splitter (PBS). Since the q-plate acts as an interface between OAM and polarization spaces, it converts the OAM-encoded information into polarization that, in a further step, we analyze with a second polarization analysis stage^30,31^. Finally, the photons are coupled into single mode fibers to ensure that only states with $\ell =0$ are detected. Our experimental setup allows thus to perform measurements of all four-qubit operators (Pauli’s matrices), consequently including full quantum state tomography (FQST). The measurement of any four Pauli operators needs in general 16 independent measurements. The witness given in Eq. (2), however, needs only 144 measurements (not 16 · 16 = 256 since the unit and *Z* operator have common eigenvectors). In strong contrast, a full quantum state tomography requires 1296 measurements.

### Experimental Results

**Figure 3:**
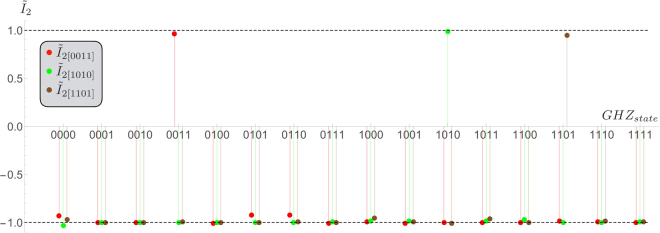
Robustness of HMGH criterion. Application of ${\tilde{I}}_{2}$ onto all generated GHZ states in the set, optimized for three different states: the twin state *ρ*_0011_ of *ρ*_0000_, *ρ*_1010_ and *ρ*_1110_. In perfect agreement with the theoretical predictions a detection is only successful in case of the matching witness. Note that for the full witness *I*_2_ both twin-states are optimally detected (in linearization the local information distinguishing the twins is lost).

In a first step we have generated all 16 GHZ basis states and measured the local observables of the witness (both using raw data and with dark counts corrections). The results are listed in the Table 1 and show a high stability among all 16 GHZ states. The averaged over all basis states is ${\tilde{I}}_{2}=0.90\pm 0.06$ (${\tilde{I}}_{2}=0.80\pm 0.05$) with (without) dark counts correction, respectively. Moreover, we tested the robustness by applying three chosen witnesses to all 16 GHZ basis states. As expected we found ${\tilde{I}}_{2} > 0$ only for those states where the basis representation matches i.e the basis representation of the state matches with the basis choice for the observable, whereas in all other cases it is clearly negative, see Fig. 3.

**Table 1:** Experimental results for the witness ${\tilde{I}}_{2}$ applied to all orthogonal basis GHZ-states. Normalization factors are omitted for brevity. A value greater than zero witnesses that the state is not $k=2/3$-separable, respectively.

GHZ State	${\tilde{I}}_{2}$ (raw data)	${\tilde{I}}_{2}$ (dark counts corr.)
|*GHZ*_0000_〉 = |*RrRr*〉 + |*LlLl*〉	0.751 ± 0.007	0.830 ± 0.007
|*GHZ*_0001_〉 = |*RrRl*〉 − |*LlLr*〉	0.765 ± 0.006	0.844 ± 0.006
|*GHZ*_0010_〉 = |*RrRl*〉 + |*LlLr*〉	0.758 ± 0.009	0.991 ± 0.009
|*GHZ*_0011_〉 = |*RrRr*〉 − |*LlLl*〉	0.871 ± 0.003	0.966 ± 0.003
|*GHZ*_0100_〉 = |*RrLr*〉 + |*LlRl*〉	0.782 ± 0.005	0.886 ± 0.005
|*GHZ*_0101_〉 = |*RrLr*〉 − |*LlRl*〉	0.722 ± 0.005	0.823 ± 0.005
|*GHZ*_0110_〉 = |*RrLr*〉 + |*LlRrl*〉	0.766 ± 0.007	0.849 ± 0.007
|*GHZ*_0111_〉 = |*RrLr*〉 + |*LlRl*〉	0.746 ± 0.006	0.830 ± 0.006
|*GHZ*_1000_〉 = |*RlRr*〉 + |*LrLl*〉	0.845 ± 0.005	0.913 ± 0.005
|*GHZ*_1001_〉 = |*RlRl*〉 − |*LrLr*〉	0.814 ± 0.008	0.957 ± 0.007
|*GHZ*_1010_〉 = |*RlRl*〉 + |*LrLr*〉	0.827 ± 0.012	0.990 ± 0.008
|*GHZ*_1011_〉 = |*RlRr*〉 − |*LrLl*〉	0.763 ± 0.006	0.838 ± 0.006
|*GHZ*_1100_〉 = |*RlLr*〉 + |*LrRl*〉	0.827 ± 0.004	0.915 ± 0.004
|*GHZ*_1101_〉 = |*RlLl*〉 − |*LrRR*〉	0.837 ± 0.006	0.950 ± 0.006
|*GHZ*_1110_〉 = |*RlLl*〉 + |*LrLr*〉	0.822 ± 0.006	0.952 ± 0.006
|*GHZ*_1111_〉 = |*RlLr*〉 − |*LrRl*〉	0.860 ± 0.005	0.928 ± 0.005

Furthermore, we have performed a full state tomography of two selected basis states (see Fig. 2b) and applied the theoretical nonlinear witness to the obtained state ${\rho }_{exp}^{{\rm{FQST}}}$, i.e. ${I}_{2}({\rho }_{exp}^{{\rm{FQST}}})$, and as well the linearized witness ${\tilde{I}}_{2}({\rho }_{exp}^{{\rm{FQST}}})$. The data are given in Table 2 and show similar results independent of the method. We checked the purity $P=Tr(({\rho }_{exp}^{{\rm{FQST}}}{)}^{2})$ of the two states and found: $P({\rho }_{0101})=0.905\pm 0.002$, $P({\rho }_{0111})=0.915\pm 0.002$. A standard maximum likelihood method has then been exploited to numerically evaluate each density operator^32^. The values are comparable and explain the deviations from the optimal value ${\tilde{I}}_{2}(GHZ)=1$. In summary, all produced states are certainly genuine multipartite entangled, i.e. there exist no bipartition via any partition of all involved degrees of freedom. Since all measured values are in good agreement by taking into account the purity of the generated states, the data clearly show the independence on the degrees of freedom chosen and local basis choices.

**Table 2:** Evaluation of the HMGH criterion *I*_2_ for two generated GHZ states. Starting from the experimental density matrix ${\rho }_{exp}^{{\rm{FQST}}}$ we evaluated the criterion ${I}_{2}({\rho }_{exp}^{{\rm{FQST}}})$ (first column) and its linearized version, Eq. (2), (second column). These two values can be compared to the values directly obtained by measuring the witness, Eq. (2), (third column).

	${I}_{2}({\rho }_{exp}^{{\rm{FQST}}})$	${\tilde{I}}_{2}({\rho }_{exp}^{{\rm{FQST}}})$	${\tilde{I}}_{2}$
*ρ*_0111_	0.986 ± 0.002	0.865 ± 0.002	0.830 ± 0.006
*ρ*_0101_	0.893 ± 0.002	0.845 ± 0.003	0.823 ± 0.005

### Entanglement properties of mixtures of GHZ states

**Figure 4:**
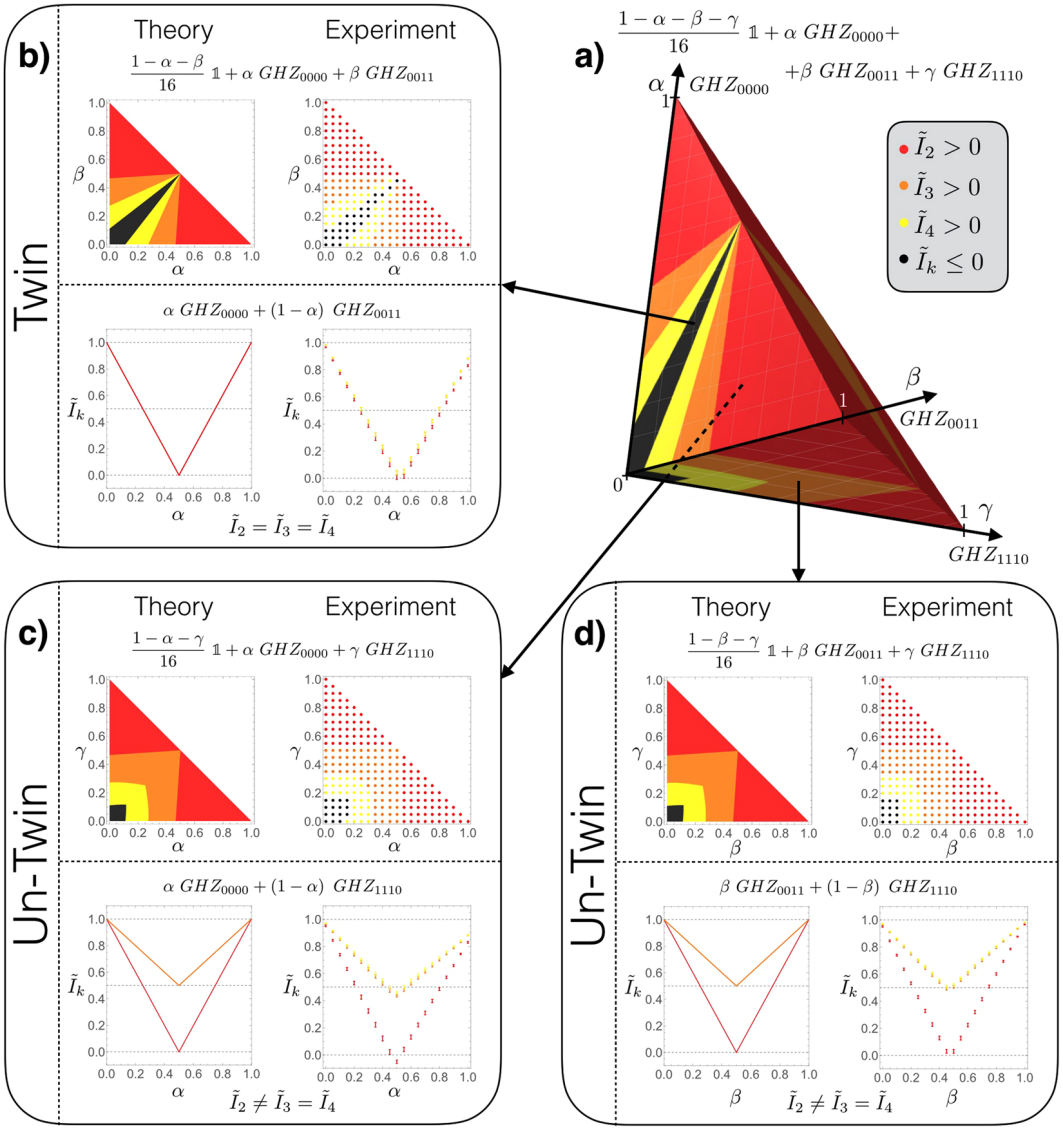
Theoretical and experimental results for GHZ mixtures. (**a**) Theoretical geometry of the mixture of three GHZ in the presence of white noise. The parameters *α* and *β* are the statistical weights of two twin GHZ (in this case *GHZ*_0000_ and *GHZ*_0011_) while *γ* is the weight of the un-twin one (*GHZ*_1110_). Red regions represent mixed states which are not bi-separable (i.e. are entangled in a multipartite sense), orange (yellow) regions correspond to states which are not *k* = 3 (*k* = 4)-separable but are bi-separable, black regions represent those states on which ${I}_{k}\le 0$ or equivalently the states are invariant under partial transpose. This peculiar geometry holds for any choice of two twin GHZ and an un-twin one. (**b–d**) Theoretical and experimental geometry for the mixtures of two GHZ with and without white noise. On the left side of each box are shown the theoretical mixtures with (on the top) and without (on the bottom) noise, while on the right are shown the corresponding experimental results. The (**b**) box shows mixtures of twin GHZ, where the equal mixture of both states results in a separable state. (**c,d**) boxes show mixtures of two pairs of un-twin GHZ having the same geometry: equal mixture of both states are bi-separable but are not *k* = 3-separable.

For revealing the local substructure of mixtures of GHZ states we considered mixtures of white noise and three GHZ states *ρ*_*i*_

5 $$\rho (\alpha ,\beta ,\gamma )=\frac{1-\alpha -\beta -\gamma }{16}{\mathbb{1}}+\alpha \,{\rho }_{1}+\beta \,{\rho }_{2}+\gamma \,{\rho }_{3}$$

where *α*, *β* and *γ* are statistical weights. Such a state can be experimentally obtained by collecting photons for each component for a time proportional to its weight in the mixture or, equivalently, by combining raw data for each component with the appropriate weights. In order to perform a complete study of the state geometry we adopted the latter approach which allows to span the whole parameters space ($\alpha ,\beta ,\gamma$). As stated in the beginning a chosen GHZ state has always exactly one geometrical twin. Without loss of generality we assume that *ρ*_1_, *ρ*_2_ are such a pair, i.e. the equal mixture of both states results in a separable state. Whereas a mixture of *ρ*_1_ with any other GHZ state *ρ*_3_ is not *k* = 3-separable. Figure 4 shows the theoretical and experimental geometry for a given choice of $\rho (\alpha ,\beta ,\gamma )$ (section (a)) and its corresponding sub-mixtures of two GHZ with (and without) white noise (sections (b-d): $\rho (\alpha ,\beta )$, $\rho (\alpha ,\gamma )$ and $\rho (\beta ,\gamma )$. This figure shows how mixtures of twin and un-twin GHZ exhibit different behaviors: mixtures of twin pairs (b) are fully separable if weights in the mixture have the same value, while this is not true if we look at mixtures of un-twin pairs (c, d) in which the states are bi-separable but not three-separable considering again mixtures having the same weights for both the states. Finally one can notice that the regions of bi-inseparability coincide for twin or un-twin mixtures, although regions of three and four-inseparability are different in the two cases. Moreover, looking separately to twin and un-twin mixtures, three and four-inseparability coincide in absence of noise, showing a different behavior when the mixture becomes noisy.

### Discussions and Outlook

We have considered states in a four-tensored Hilbert-space where each subspace is described by two dimensions which we physically achieved by manipulating the polarization and orbital momentum degrees of freedom of two photons. Producing a complete set of orthogonal GHZ states and their detection via entanglement witnesses showed a high quality in always achieving states with same entanglement properties but locally different geometries. Local differences are important when mixing those states. In particular we proved experimentally that among the 16 GHZ states each GHZ state has always a twin that when mixed with equal weights gives a fully separable state. In opposition any other balanced mixtures of GHZ states destroy genuine multipartite entanglement, but not any other type of entanglement. Certainly, this local information between orthogonal basis states is relevant for any experimental setup since it is experimentally accessible without quantum state tomography and as we show can be designed to generate particular types of entanglement paving the way to new applications involving topics such as device-independence witnesses^33^, secret sharing protocols based on the mixtures of GHZ states^34^ and for quantum algorithms exploring different types of multipartite entanglement^35,36^.

### Appendix

In ref.^37^. it was proven that any state *ρ* (mixed or pure) that is *k*-separable has to satisfy ${I}_{k}(\rho )\le 0$ where for a 16 × 16 matrix *ρ* the functions (optimized for the state in Eq. 1 of the main text) read explicitly

$$\begin{array}{c}{I}_{k\mathrm{=2}}(\rho )=\mathrm{2|}{\rho }_{\mathrm{1,16}}|-(\sqrt{{\rho }_{\mathrm{2,2}}\,{\rho }_{\mathrm{15,15}}}+\sqrt{{\rho }_{\mathrm{3,3}}\,{\rho }_{\mathrm{14,14}}}\\ \quad \quad \quad \,\,\,\,\,+\sqrt{{\rho }_{\mathrm{4,4}}\,{\rho }_{\mathrm{13,13}}}+\sqrt{{\rho }_{\mathrm{5,5}}\,{\rho }_{\mathrm{12,12}}}+\sqrt{{\rho }_{\mathrm{6,6}}\,{\rho }_{\mathrm{11,11}}}\\ \quad \quad \quad \,\,\,\,\,+\sqrt{{\rho }_{\mathrm{7,7}}\,{\rho }_{\mathrm{10,10}}}+\sqrt{{\rho }_{\mathrm{8,8}}\,{\rho }_{\mathrm{9,9}}}),\\ {I}_{k\mathrm{=3}}(\rho )=\mathrm{2|}{\rho }_{\mathrm{1,16}}|-({({\rho }_{\mathrm{2,2}}{\rho }_{\mathrm{3,3}}{\rho }_{\mathrm{4,4}}{\rho }_{\mathrm{13,13}}{\rho }_{\mathrm{14,14}}{\rho }_{\mathrm{15,15}})}^{\frac{1}{6}}\\ \quad \quad \quad \,\,\,\,\,+{({\rho }_{\mathrm{2,2}}{\rho }_{\mathrm{5,5}}{\rho }_{\mathrm{6,6}}{\rho }_{\mathrm{11,11}}{\rho }_{\mathrm{12,12}}{\rho }_{\mathrm{15,15}})}^{\frac{1}{6}}\\ \quad \quad \quad \,\,\,\,\,+{({\rho }_{\mathrm{2,2}}{\rho }_{\mathrm{7,7}}{\rho }_{\mathrm{8,8}}{\rho }_{\mathrm{9,9}}{\rho }_{\mathrm{10,10}}{\rho }_{\mathrm{15,15}})}^{\frac{1}{6}}\\ \quad \quad \quad \,\,\,\,\,+{({\rho }_{\mathrm{3,3}}{\rho }_{\mathrm{5,5}}{\rho }_{\mathrm{7,7}}{\rho }_{\mathrm{10,10}}{\rho }_{\mathrm{12,12}}{\rho }_{\mathrm{14,14}})}^{\frac{1}{6}}\\ \quad \quad \quad \,\,\,\,\,+{({\rho }_{\mathrm{3,3}}{\rho }_{\mathrm{6,6}}{\rho }_{\mathrm{8,8}}{\rho }_{\mathrm{9,9}}{\rho }_{\mathrm{11,11}}{\rho }_{\mathrm{14,14}})}^{\frac{1}{6}}\\ \quad \quad \quad \,\,\,\,\,+{({\rho }_{\mathrm{4,4}}{\rho }_{\mathrm{5,5}}{\rho }_{\mathrm{8,8}}{\rho }_{\mathrm{9,9}}{\rho }_{\mathrm{12,12}}{\rho }_{\mathrm{13,13}})}^{\frac{1}{6}}),\\ {I}_{k\mathrm{=4}}(\rho )=\mathrm{2|}{\rho }_{\mathrm{1,16}}|-2{({\rho }_{22}{\rho }_{33}{\rho }_{55}{\rho }_{88}{\rho }_{99}{\rho }_{\mathrm{12,12}}{\rho }_{\mathrm{14,14}}{\rho }_{\mathrm{15,15}})}^{\frac{1}{8}}\mathrm{.}\end{array}$$
